# Prevalence and Associated Factors of Self‐Medication Among Adults With Hypertension in Central Uganda: A Cross‐Sectional Study

**DOI:** 10.1155/ijhy/9749228

**Published:** 2026-07-29

**Authors:** Charity Kakuru, Julius Kyomya, Abraham Muhwezi, Tom Murungi, Henry Ochola, Eleanor Turyakira, Richard Migisha, Peter Chris Kawungezi

**Affiliations:** ^1^ Department of Community Health, Faculty of Medicine, Mbarara University of Science and Technology, Mbarara, Uganda, must.ac.ug; ^2^ Department of Pharmacy, Faculty of Medicine, Mbarara University of Science and Technology, Mbarara, Uganda, must.ac.ug; ^3^ Department of Midwifery, Faculty of Nursing and Midwifery, Lira University, Lira, Uganda, lirauni.ac.ug; ^4^ Department of Obstetrics and Gynecology, Faculty of Medicine, Mbarara University of Science and Technology, Mbarara, Uganda, must.ac.ug; ^5^ Uganda Public Health Fellowship Program, Uganda National Institute of Public Health, Kampala, Uganda; ^6^ Department of Physiology, Mbarara University of Science and Technology, Mbarara, Uganda, must.ac.ug

**Keywords:** antihypertensive agents, chronic care, hypertension, low- and middle-income country, self-medication

## Abstract

**Background:**

Self‐medication is a widespread practice among individuals with chronic conditions such as hypertension. It can lead to poor blood pressure control and an increased risk of complications. However, little is known about its prevalence and associated factors among patients with hypertension in Uganda. This study aimed to determine the prevalence and associated factors of self‐medication among adults with hypertension at Masaka Regional Referral Hospital (MRRH), Central Uganda.

**Methods:**

A cross‐sectional study was conducted during August and September 2024 among patients with known hypertension aged ≥ 18 years attending the MRRH Hypertension Clinic. Systematic sampling was used to recruit participants. Data were collected using a structured questionnaire, capturing sociodemographic and clinical characteristics. We defined self‐medication as the use of antihypertensive medicines by individuals to treat hypertension without a formal prescription or guidance from a healthcare professional within the last 90 days before the survey. Modified Poisson regression was used to identify factors associated with self‐medication.

**Results:**

We enrolled 192 participants, with a mean age of 58 (±14) years; 143 (75%) were female. The prevalence of self‐medication with antihypertensive drugs was 47% (95% confidence interval [CI] = 40%–54%). Factors associated with self‐medication were low monthly income (< 2,00,000 UGX; aPR = 2.00; 95% CI: 1.04–3.86), 20 medication gap (adjusted Prevalence Ratio [aPR] = 1.46; 95% CI: 1.01–2.11), and familial influence (aPR = 1.41; 95% CI: 1.04–1.92).

**Conclusion:**

The prevalence of self‐medication among hypertensive patients at MRRH was high. Self‐medication was associated with low monthly income, medication gap, and familial pressure. The findings suggest that interventions addressing financial barriers, medication availability, and family influence warrant further evaluation as potential strategies to reduce self‐medication.

## 1. Background

Hypertension affects approximately one‐third of adults aged 30–79 years worldwide and is the principal risk factor for cardiovascular diseases, the leading cause of death globally [1]. Low‐ and middle‐income countries shoulder nearly two‐thirds of this burden [2], yet only 42% of the hypertensive individuals are diagnosed and treated [2]. Sub‐Saharan Africa (SSA) has a high prevalence of hypertension, with East African nations exhibiting one of the highest rates worldwide [3–5]. In Uganda, a recent national survey estimated hypertension prevalence at 31.5% [6], highlighting its status as a critical public health challenge in this resource‐constrained context.

Self‐medication, the use of drugs without professional guidance, affects 11.7%–92% of people worldwide, with up to 80% of medications obtained without a prescription in developing countries [7–13]. It is regarded as one of the major health and socioeconomic challenges [14]. The practice of self‐medication is common in Uganda, with a 52% prevalence among adults [15]. These high rates of self‐medication pose risks of drug misuse, adverse reactions, and an extra burden on health services. Patients with chronic conditions such as hypertension are particularly prone to self‐medication, often turning to over‐the‐counter and alternative therapies [16, 17]. Inappropriate self‐medication can precipitate harmful drug interactions, mask underlying pathology, and delay proper diagnosis [9, 18]. For individuals with hypertension, these practices can lead to suboptimal blood pressure control and increase the risk of myocardial infarction, stroke, and hypertensive heart disease [19, 20].

In Uganda and other low‐ and middle‐income countries, self‐medication is fueled by overburdened health systems, insufficient numbers of trained clinicians and pharmacists, suboptimal prescribing practices, and weak regulatory enforcement of prescription‐only drugs [7, 8, 21]. The high prevalence of self‐medication with prescription drugs in these countries is particularly concerning due to less stringent regulation around medication use [18]. With the high prevalence of hypertension in Central Uganda (34.3%) [6] and the high prevalence of self‐medication [15], it is crucial to understand the self‐medication practices among patients with hypertension. Moreover, little is known about the prevalence and associated factors of self‐medication among known hypertensive patients in central Uganda. Therefore, we conducted this study to determine the prevalence and associated factors of self‐medication among patients with known hypertension in Masaka City, central Uganda.

## 2. Materials and Methods

### 2.1. Study Design and Setting

This study employed a cross‐sectional design during August and September 2024. The study was conducted at the hypertension clinic of Masaka Regional Referral Hospital (MRRH). The hospital serves as the referral center for Masaka and the greater Central region, including Bukomansimbi, Kalangala, Kalungu, Kyotera, Lwengo, Lyantonde, Masaka, Rakai, and Ssembabule. The hospital runs an active hypertension clinic every Thursday, with a capacity of 300 active clients as of December 31, 2023, and receives an average of 75 clients each clinic day. In central Uganda, 34.3% of the adults have hypertension [6], of which 73.3% have their blood pressure uncontrolled [22]. Since self‐medication is a common practice among adults [15], it is crucial to evaluate these practices among patients with hypertension at a tertiary facility in central Uganda.

### 2.2. Study Population

We recruited adults (≥ 18 years) with a confirmed diagnosis of hypertension who were receiving care at MRRH. Pregnant women with pregnancy‐related hypertensive disorders, such as pre‐eclampsia, were excluded from the study as these conditions are often temporary and may not accurately represent chronic hypertension. Additionally, patients in hypertensive crisis with stroke, Myocardial infarction, or encephalopathy that could affect comprehension were excluded.

### 2.3. Sample Size and Sampling Procedure

The sample size was estimated using the Kish and Leslie formula, *n* = (*Z* × *p* × *q*)/*d* ^2^ [23], with *Z* = 1.96 (95% confidence interval [CI]), *p* = 0.54 (estimated self‐medication prevalence among adult patients with hypertension from Ghana) [24], *q* = 0.46, and *d* = 0.05, yielding *n* ≈ 382.

Because the actual population of patients with hypertension at Masaka Referral Hospital was 300, the sample size was adjusted using Daniel’s (1999) sample size adjustment formula [25], yielding a sample of 168 participants. The study added 10% of the sample size to cater for a potential nonresponse rate. Thus, the adjusted sample size of the study was 185 participants.

We employed a systematic sampling method to select participants for this study. With a sample size of 185 and a population size of 300, a sampling interval of two was used. To initiate the sampling, the first participant was selected by simple random sampling from the first two eligible patients. Thereafter, every 2nd eligible patient was approached for participation and administered a questionnaire upon providing informed consent until the required sample size was achieved.

### 2.4. Data Collection Methods and Tools

The study used a researcher administered questionnaire developed from similar previous studies [24, 26]. The tool was pretested with 10 patients diagnosed with hypertension at Mbarara Regional Referral Hospital to ensure clarity, appropriateness and contextual relevance. Results of the pretest were later used to modify the tool. The questionnaire was translated from English to Luganda, the local language, to ensure optimal comprehension and accurate collection. The tool captured information on (1) sociodemographic characteristics (gender, age, monthly income, level of education, knowledge, residence address, religion, marital status, and occupation), (2) self‐medication practices (frequency of self‐medication, type of drugs used, and reasons for self‐medication), (3) sociocultural factors (family values, gender roles and values, cultural beliefs and practices, social support, and peer influence), (4) health system factors (accessibility, provider availability, trust, and availability of over‐the‐counter drugs), and (5) policy factors (drug price regulation, formal and informal health structures, and advertising and promotion).

### 2.5. Study Variables

The outcome variable in this study was self‐medication, defined as the use of antihypertensive medicines to manage hypertension without a formal prescription or guidance from a healthcare professional at any point in the last 90 days. This was measured using a dichotomous response (yes/no) indicating whether a participant had ever engaged in self‐medication or not.

We chose a 90‐day recall period for assessing self‐medication to balance accuracy and relevance. This timeframe reduces recall bias and minimizes misclassification, as participants were more likely to accurately remember recent medication practices compared to longer periods. It is also sufficiently long to capture intermittent or irregular self‐medication behaviors.

The predictor variables in this study were categorized into four broad domains: individual, social and cultural, health system, and policy‐related factors. Individual factors (Items Q1–Q13) included sociodemographic and personal characteristics such as gender, age, monthly income, level of education, knowledge about hypertension and medication use, residence, religion, marital status, occupation, and family size. Social and cultural factors (Items Q27–Q29) encompassed influences such as family values and support systems, social networks, prevailing gender roles, cultural beliefs and practices regarding illness and treatment, and peer influence on medication behaviors. Medication gap was defined as a self‐reported period in which a participant missed prescribed antihypertensive medication because the medicines ran out before the next scheduled clinic visit. This variable was assessed using a dichotomous question (Yes/No). Familial influence was defined as participant‐reported advice, recommendation, or pressure from family members to take antihypertensive medication without professional guidance. This variable was assessed using a dichotomous question and coded as 1 = Yes and 0 = No.

Health system factors (Items Q30–Q36) covered issues related to healthcare access and quality, including geographical accessibility, availability of healthcare providers, patient trust in the health system, the availability of over‐the‐counter drugs, use of technology and telemedicine, consistent supply of antihypertensive medications at health facilities, and access to accurate healthcare information. Finally, policy factors (Items Q37‐Q38) included regulatory and structural elements such as pharmacy licensing and drug control mechanisms, drug price regulation, the presence and influence of formal and informal health systems, pharmaceutical advertising and promotion, and consumer protection laws that govern medicine use and availability.

### 2.6. Data Collection Procedure

Participants were gathered at the hypertension clinic of MRRH, where every second patient on the appointment list was invited to participate through systematic random sampling. Two trained research assistants, fluent in both English and Luganda, conducted all interviews in private consultation rooms to ensure privacy. After obtaining written informed consent, each participant completed a 30‐min session in which the assistant guided them through a structured questionnaire. We employed Kobo Collect, an open‐source Android application, to record responses directly on tablets. Interviewers probed for clarity whenever answers were ambiguous, corrected data‐entry errors on the spot, and securely synced encrypted records at the end of each day to maintain data integrity and participant privacy.

### 2.7. Data Management and Analysis

All data captured through Kobo Collect was encrypted and securely synchronized over a password‐protected central server (https://ee.kobotoolbox.org/x/BcRSyCdv). Data were downloaded into an Excel spreadsheet, cleaned, and transferred to SPSS Version 27 for descriptive analysis. Modified Poisson regression with robust standard errors was conducted using Stata Version 17.

We employed both descriptive and inferential statistics to determine the prevalence and factors associated with self‐medication among patients with known hypertension. The prevalence of self‐medication was determined as the proportion of patients with known hypertension (denominator) who reported self‐medication (numerator) and expressed as a percentage with a corresponding 95% CI. Continuous variables were summarized using the mean and standard deviation if normally distributed, and the median with interquartile range if skewed. Modified Poisson regression with robust standard errors was used to determine factors associated with self‐medication among patients with hypertension. Initially, all variables with a *p* value <  0.2 in the bivariate analysis were included in the multivariable model. However, a backward elimination approach was applied to refine the final model. Three variables (cultural influence, self‐reported health status, and gender) that met the initial inclusion criteria were excluded from the final analysis. These variables were dropped because they were not statistically significant in the multivariable model (*p* > 0.40) and did not improve the model fit based on the Akaike information criterion (AIC). Furthermore, the exclusion of cultural influence resolved collinearity issues with family influence. Model diagnostics were performed in Stata Version 17. We assessed goodness‐of‐fit using deviance and Pearson chi‐square statistics. A link test was conducted to evaluate model specification; the squared linear predictor was not statistically significant (*p* = 0.722), indicating that the model was correctly specified. Statistical significance was set at *p* < 0.05, and results were reported as adjusted Prevalence Ratios (aPRs) with their 95% CIs.

### 2.8. Ethical Considerations

This study was conducted in accordance with the ethical principles of the Declaration of Helsinki and relevant national guidelines and received ethical approval from the ethics committee of Mbarara University of Science and Technology (IRB No: MUST‐REC‐2024‐1613). Additionally, administrative clearance was obtained from the director of MRRH to conduct the study at the facility. Written informed consent was obtained from the study participants before the interviews, and they were interviewed in private and quiet clinical rooms. All personal identification information was made anonymous by using unique participant codes to maintain confidentiality. In addition, raw and cleaned data access was restricted to the principal investigator and the research team members.

## 3. Results

### 3.1. Participants

In this study, 200 participants with a confirmed diagnosis of hypertension receiving care at the Hypertension Clinic of MRRH were approached for enrollment in this study. However, 3/200 (1.5%) participants declined to participate in the study, and 5/200 (2.5%) participants were in hypertensive crisis and thus unable to participate. Thus, 96% (192/200) were enrolled into the study.

### 3.2. Sociodemographic Characteristics of the Study Participants

The median age of participants was 58 years (IQR: 50–69), with most of them, 89/192 (46.4%), being aged 40–59 years. The majority of the participants, 143/192 (74.5%), were female. Most of the participants, 96/192 (50.0%), were either married or cohabiting. The majority of the participants, 160/192 (83.3%), reported an average monthly income of less than 2,00,000 Uganda shillings (Table 1).

**TABLE 1:** Sociodemographic characteristics of the study participants in a study assessing the prevalence and associated factors of self‐medication among adults with known hypertension at Masaka Regional Referral Hospital, Masaka City, Uganda, 2024, *N* = 192.

Variable	Frequency (*n*), *N* = 192	(%)
Age (years)^∗^		
19–39	16	8.3
40–59	89	46.4
60+	87	45.3
Gender		
Female	143	74.5
Male	49	25.5
Marital status		
Single	32	16.7
Married/cohabiting	96	50
Divorced/separated/widowed	64	33.3
Occupation		
Unemployed	35	18.2
Self‐employed	106	55.2
Formal employment	51	26.6
Education level		
No formal education	35	18.2
Primary	106	55.2
Secondary	39	20.3
Tertiary	12	6.3
Religion		
Anglican/born again	42	21.9
Catholic	113	58.8
Moslem	37	19.3
Average monthly income (UGX)^∗∗^		
< 2,00,000	160	83.3
≥ 200,000	32	16.7
Estimated distance from the HTN clinic (km)		
≤ 5 km	106	55.2
> 5 km	86	44.8
Transport cost for each HTN clinic visit (UGX)		
< 5000	64	33.3
5000–10,000	55	28.6
> 10,000	73	38.0

*Note:* UGX‐Uganda Shillings.

Abbreviation: km, kilometer.

^∗^Median (IQR) = 58 (50‐69).

^∗∗^Currency conversion: 3700 UGX ≈ 1 USD.

### 3.3. Prevalence of Self‐Medication

Out of the 192 participants, 90 reported self‐medication with antihypertensive medication in the last 90 days, giving a prevalence of 47% (95% CI: 40%–54%).

Factors associated with self‐medication with antihypertensives among patients with known hypertension at MRRH.

In the multivariable modified Poisson regression, three factors remained independently associated with self‐medication. After adjusting for other variables, participants with an average monthly income of less than 2,00,000 Ugandan shillings showed a twofold higher prevalence of self‐medication (aPR = 2.00; 95% CI: 1.04–3.86, *p* = 0.039) compared to those earning > 2 00,000 UGX. Participants with a medication gap had a 46% higher prevalence of self‐medication (aPR = 1.46; 95% CI: 1.01–2.11, *p* = 0.045) compared to those with no gaps. Similarly, participants reporting familial influence showed a 41% higher prevalence of self‐medication with antihypertensives (aPR = 1.41; 95% CI: 1.04–1.92, *p* = 0.026) compared to those with no family influence (Table 2).

**TABLE 2:** Bivariate and multivariable modified Poisson regression of factors associated with self‐medication among patients with known hypertension in Masaka Regional Referral Hospital, 2024.

Variable	Self‐medication							
	No (*n*), *N* = 102	(%)	Yes (*n*), *N* = 90	(%)	cPR	(95% CI)	aPR	(95% CI)
Gender								
Female	67	(65.7)	76	(84.4)	1.9	(1.2–3.0)		
Male	35	(34.3)	14	(15.6)	Ref			
Duration with HTN (years)								
< 5	57	(55.9)	41	(45.6)	Ref		Ref	(0.8–1.6)
5–10	20	(19.6)	25	(27.8)	1.3	(0.9–1.9)	1.16	(0.71–1.40)
> 10	25	(24.5)	24	(26.7)	1.2	(0.82–1.63)	0.99	
Marital status								
Unmarried	45	(44.1)	51	(56.7)	1.3	(0.9–1.8)	1.12	(0.83–1.52)
Married	57	(55.9)	39	(43.3)	Ref		Ref	
Occupation								
Unemployed	33	(32.4)	36	(40.0)	2.2	(0.9–5.4)	1.49	(0.57–3.85)
Self‐employed	56	(54.9)	50	(55.6)	2.0	(0.8–4.8)	1.76	(0.69–4.5)
Formal employment	13	(12.7)	4	(4.4)	Ref		Ref	
Education level								
No formal education	16	(15.7)	19	(21.1)	2.2	(0.8–26.1)	1.17	(0.33–2.82)
Primary level	57	(55.9)	49	(54.5)	1.8	(0.7–25.0)	0.97	(0.43–3.68)
Secondary level	20	(19.6)	19	(21.1)	1.9	(0.7–5.5)	1.25	
Tertiary level	9	(8.8)	3	(3.3)	Ref		Ref	
Average monthly income (UGX)^∗^								
< 2,00,000	77	(75.5)	83	(92.2)	2.4	(1.2–4.6)	**2.00**	**(1.04–3.86)**
≥ 2,00,000	25	(24.5)	7	(7.8)	Ref		Ref	
Educated on medication								
No	34	(33.3)	52	(57.8)	1.8	(0.9–3.4)	1.37	(0.72–2.60)
Yes	51	(50.0)	31	(34.4)	1.6	(0.8–3.1)	1.16	x
Don’t remember	17	(16.7)	7	(7.8)	Ref		Ref	
Medication gap								
No	43	(42.2)	21	(23.3)	Ref		Ref	
Yes	59	(57.8)	69	(76.7)	1.6	(1.1–2.4)	**1.46**	**(1.01–2.11)**
Health status (self‐reported)								
Poor	32	(31.4)	37	(41.1)	1.2	(0.9–1.7)		
Good	70	(68.6)	53	(58.9)	Ref			
Familial influence								
No	74	(72.6)	43	(47.8)	Ref		Ref	
Yes	28	(27.4)	47	(52.2)	1.7	(1.3–2.3)	**1.41**	**(1.04–1.92)**
Cultural influence								
No	84	(82.4)	62	(68.9)	Ref			
Yes	18	(17.6)	28	(31.1)	1.4	(1.1–1.9)		
Adverts								
No	48	(47.1)	25	(27.8)	Ref		Ref	
Yes	54	(52.9)	65	(72.2)	1.6	(1.1–2.3)	1.24	(0.87–1.77)
Follow‐up								
Immediate (1–3 weeks)	23	(22.5)	9	(10.0)	Ref		Ref	
Routine (4 weeks)	68	(66.7)	71	(78.9)	1.8	(1.0–3.2)	1.57	(0.87–2.82)
Extended (> 4 weeks)	23	(10.8)	10	(11.1)	1.7	(0.8–3.5)	1.38	(0.67–2.84)

*Note:* UGX: Ugandan shillings. The bolded values are showing variables that remained significant during multivariable analysis.

Abbreviations: aPR, adjusted Prevalence Ratios; CI, confidence interval; cPR, crude Prevalence Ratios.

^∗^Currency conversion: 3700 UGX ≈ 1 USD, HTN: hypertension.

## 4. Discussion

This study aimed to assess the prevalence and factors associated with self‐medication among adults with known hypertension at MRRH, Uganda. Close to half of the participants reported self‐medication with antihypertensive drugs. Factors associated with self‐medication were low monthly income, medication gap, and familial influence.

The study found that nearly half of the hypertensive patients self‐medicate with antihypertensive agents, a practice likely driven by fragmented access to supervised care, extended clinic wait times, frequent public‐sector stock‐outs, and out‐of‐pocket expenses [27, 28]. This prevalence mirrors the 55.6% (within the 95% CI) reported by a systematic review in Uganda [15], yet far surpasses the 22.2% documented among patients at Kiruddu Hospital in Uganda [26]. The prevalence in our study is lower than the 60.1% obtained among hypertensive patients in Abidjan [29]. Additionally, our prevalence remains below the 93.8% [30] and 96.7% [31] observed among medical students in Uganda. The differences in prevalence may be attributed to variations in study populations and the focus on self‐medication with antibiotics among medical students. The high self‐medication prevalence reflects deep‐rooted challenges within the healthcare infrastructure, most notably limited access to supervised care, prolonged waiting times at clinics, frequent stock‐outs of essential medications, and the financial burden of out‐of‐pocket payments [32]. These systemic barriers not only encourage self‐medication but also undermine continuity of care and long‐term blood pressure control, increasing the risk of complications such as stroke, renal failure, and cardiovascular events. These findings suggest that improving patient–provider communication, ensuring consistent drug availability, and developing local guidance on unsupervised antihypertensive use may be important areas for future intervention studies.

Our findings show that participants with a monthly income < 2,00,000 UGX (USD 54) had a twofold higher prevalence of self‐medication compared to those earning more, highlighting economic hardship as a principal driver of unsupervised antihypertensive use. This finding aligns with observations among older adults in China [33] and similar findings in India [34] and Vietnam [35], suggesting a consistent pattern across LMICs. The observed association between low income and self‐medication suggests that interventions addressing financial barriers, such as community‐based income‐generating activities or patient savings schemes, may be worth exploring in future studies [36]. Additionally, service delivery approaches such as mobile outreach clinics or community health worker–led medication distribution could be explored as potential strategies to reduce access barriers [37]. Promoting hypertensive patient Savings and Credit Cooperative Organization (SACCO) initiatives may also represent a potential approach to improving financial access to care.

Participants who had a medication gap as a result of medications running out before their next clinical visit had a higher prevalence of self‐medication compared to those with sufficient medication. A plausible explanation is that interruptions in medication access may heighten concerns about missed doses and potential deterioration in the health status, prompting individuals to seek alternative, informal sources of treatment, including borrowing medications or purchasing them over the counter without professional guidance [15]. This is almost identical to findings from a Kenyan study that identified limited medicine supply in hospitals and prolonged intervals between follow‐up appointments as major drivers of self‐medication [38]. In Uganda, the frequent stock‐outs of medicines in public facilities, as reported by the national assessment of the health supply chain, could force patients to resort to alternative means of acquiring medication [32, 39]. Self‐medication with antihypertensives without a clinician’s medical review can lead to missed assessments of underlying conditions, poor blood pressure control, and potential adverse effects [40]. These findings suggest the need to improve the supply chain systems of public health facilities, institute routine screening for self‐medication practices, and optimize follow‐up schedules to reduce dependence on self‐medication.

In this study, participants who reported familial influence had a higher prevalence of self‐medication compared to those who did not. This could be explained by the fact that family members often serve as the first line of health advice, sharing their own experiences and remedies based on personal or cultural beliefs, and patients tend to follow this guidance [41]. Moreover, a previous study among black hypertensive patients from Abidjan reported that almost 90% of the patients reported being influenced by family members to self‐medicate [29]. Our findings are also consistent with findings in northern Uganda [27] and Nigeria [42], where advice from family was associated with self‐medication behavior. Similarly, a study in rural Colombia also noted that family can negatively influence the patient and lead to self‐medication [43]. This suggests the need for community‐based education programs targeting families about the dangers of self‐medication with antihypertensive medication. Providing families with accurate medical information could be a vital intervention in curbing self‐medication practices. Because this was a cross‐sectional study, the proposed strategies should be interpreted as policy‐relevant implications rather than evidence‐based interventions.

This study has several limitations that should be considered when interpreting its findings. First, the study was conducted at a single regional referral hospital, which may limit the generalizability of the results to other regions of Uganda or similar settings. Although the study did explore participants’ reasons for self‐medication, these were not examined in depth, which may limit a comprehensive understanding of the underlying motivations and other drivers of self‐medication. In addition, while we used a 90‐day recall period to reduce recall bias and improve temporal precision, there remains a possibility that self‐reported data may have led to slight overestimation of prevalence. Finally, the cross‐sectional design limited causal inference and is subject to the influence of unmeasured confounders.

## 5. Conclusion

The prevalence of self‐medication among hypertensive patients at MRRH was high. Self‐medication was associated with low monthly income, medication gap, and familial pressure. The findings suggest that interventions addressing financial barriers, medication availability, and family influence may help reduce self‐medication practices. These strategies warrant further evaluation in interventional or longitudinal studies. In addition, future researchers should conduct multicenter studies to validate the identified factors across a broader population.

**Nomenclature**
MRRH: Masaka Regional Referral Hospital
AOR: Adjusted Odds Ratio
SM: Self‐medication
CVD: Cardiovascular diseases
COR: Crude odds ratio
HTN: Hypertension
OTC: Over the counter
POMs: Prescription‐only medications
SSA: Sub‐Saharan Africa
SPSS: Statistical Package for Social Sciences
SACCO: Savings and Credit Cooperative Organization
WHO: World Health Organization

## Author Contributions

C.K., A.M., P.C.K., J.K., and T.M. participated in the conception, design, analysis, and interpretation of the study findings and wrote the draft manuscript. H.O. participated in the analysis and interpretation of the study findings. R.M. and E.T. reviewed the report and the manuscript to ensure intellectual content and scientific integrity.

## Funding

This study was conducted without dedicated financial support or grants from any external funding agency.

## Disclosure

All authors have read and approved the final manuscript.

## Ethics Statement

This study was conducted in accordance with the ethical principles of the Declaration of Helsinki and relevant national guidelines and received ethical approval from the Ethics Committee of Mbarara University of Science and Technology (IRB No: MUST‐REC‐2024‐1613). Additionally, administrative clearance was obtained from the director of Masaka Regional Referral Hospital to conduct the study at the facility. Written informed consent was obtained from the study participants before the interviews, and they were interviewed in private and quiet clinical rooms. All personal identification information was made anonymous by using unique participant codes to maintain confidentiality. In addition, raw and cleaned data access was restricted to the principal investigator and the research team members.

## Consent

Please check the Ethics Statement.

## Conflicts of Interest

The authors declare no conflicts of interest.

## Data Availability

All materials used in this study are available from the corresponding author upon reasonable request.
